# Detecting delirium in patients with acute stroke: a systematic review of test accuracy

**DOI:** 10.1186/s12883-019-1547-4

**Published:** 2019-12-02

**Authors:** Irene Mansutti, Luisa Saiani, Alvisa Palese

**Affiliations:** 1 Department of Biomedicine and Prevention, University of Tor Vergata, Rome, Italy; 2 Department of Diagnostics and Public Health, University of Verona, Verona, Italy; 3 Department of Medical Science, University of Udine, Viale Ungheria 20, 33100 Udine, Italy

**Keywords:** Instruments, Intracerebral Haemorrhage, Ischaemic stroke, Post-stroke delirium, Tools, Sensitivity, Specificity, Systematic review

## Abstract

**Background:**

Patients with acute stroke are particularly vulnerable to delirium episodes. Although delirium detection is important, no evidence-based recommendations have been established to date on how these patients should be routinely screened for delirium or which tool should be used for this purpose in this population. Therefore, the aim of this study was to identify delirium screening tools for patients with acute stroke and to summarise their accuracy.

**Methods:**

Following the Preferred Reporting Items for Systematic Reviews and Meta-Analyses guidelines, a systematic search of Medline, CINAHL and Scopus databases was performed to include: (a) diagnostic test accuracy studies; (b) evaluating tools detecting delirium among patients with acute stroke; (c) written in English; (d) published up to September 2018. The included studies were assessed in their quality by using the Quality Assessment of Diagnostic Accuracy Studies-2.

**Results:**

A total of four studies have been performed to date in the field with a variable quality for the methodology used and documentation of the accuracy of mainly two tools, as (1) the 4-Assessment Test for delirium (4AT), reporting a range of sensitivity from 90.2 to 100% and a specificity from 64.5 to 86%; and (2) the Confusion Assessment Method-Intensive Care Unit (CAM-ICU) showing a sensitivity of 76% (95% Confidence of Interval [CI] 55–91) and a specificity of 98% (95%CI 93–100). Other tools have been studied as: The Abbreviated Mental Test-10, the Abbreviated Mental Test short form, the Clock Drawing Test, the Cognitive Examination derived from the National Institutes of Health Stroke Scale and the Glasgow Coma Scale. Moreover, the use of a single question—namely, ‘Does this patient have cognitive issues?’ as answered by the multidisciplinary team—has been subjected to a validation process.

**Conclusions:**

To date a few primary studies have been published to test the accuracy of tools in their ability to detect post-stroke delirium; among those available, the 4AT and the CAM-ICU tools have been mostly studied. Research has just started to add evidence to the challenge of detecting and usefully assessing newly-acquired delirium among stroke patients: therefore, more studies are needed to improve the knowledge and allow a robust selection of the most useful tool to use in this population.

## Background

Delirium is a complex syndrome characterised by disturbances in attention and awareness, associated with cognitive alterations (e.g., memory, language, perception)—which can emerge in hours or days—and tends to fluctuate in severity over time [1]. Delirium prevalence has been estimated at around 30% in hospitalised medical patients [2], and its occurrence rate per admission has been documented to vary from 10 to 31% [3], reaching > 50% among frail elderly patients [4].

Although patients with acute stroke are particularly vulnerable to the development of this clinical complication [5, 6], the occurrence of delirium in this population is difficult to study because of the challenges in its detection [7, 8], while risk factors, as well as short- and long- term outcomes, have been studied more often [9], including increased functional impairments, cognitive decline, length of in-hospital stay and mortality rates [4].

Aiming at preventing delirium occurrence and at minimizing its negative consequences, several clinical guidelines have been developed to date e.g., [10, 11] which generally all recommend early detection of delirium as the basis to tailor specific interventions. Some recommendations are also available regarding patients with stroke [12], although rarely incorporated into stroke care; moreover, what tool should be used in this field is still an unresolved issue, challenging clinicians in their attempts to detect delirium early in this target population [13, 14].

To date only one systematic review has been published on how delirium should be screened in patients with acute stroke by summarising evidence available on sensitivity, specificity, positive and negative predictive values [13]. However, among the 20 observational studies included in the above-mentioned systematic review, none of them was aimed at evaluating tool accuracy properties (e.g., sensitivity, specificity, positive and negative predictive values) among patients with acute stroke. In addition to the Diagnostic and Statistical Manual of Mental Disorders criteria (DSM), other tools have emerged for use in practice, such as the Confusion Assessment Method (CAM), the Delirium Rating Scale (DRS), the Mini Mental State Examination (MMSE) and the Organic Brain Syndrome scale (OBS scale). However, these tools have not been validated in this specific target population [13]. Furthermore, Carin-Levy et al. [13] highlighted the heterogeneity of the methods used in delirium detection across studies, which could also explain the wide differences of documented delirium occurrence [13]. Therefore, with the purpose of updating the available review [13], as well as to summarise the evidence available on tools detecting delirium among patients with acute stroke according to the documented accuracy properties, a systematic review has been performed.

## Methods

### Aims

This study aimed to identify delirium screening tools for patients with acute stroke and to summarise their accuracy properties. Specifically, review aims were to (a) highlight the quantity and quality of research available on accuracy properties in the target population; (b) investigate the heterogeneity of test accuracy in the included studies; (c) identify gaps in the evidence available and determine where further research is required.

### Study design

A systematic review of the literature was performed in accordance to the Preferred Reporting Items for Systematic Reviews and Meta-Analysis (PRISMA) guidelines [15]; moreover, the protocol guidance concerning test accuracy systematic reviews on problems that have a cognitive focus, was followed [16].

### Literature search

Medline, the Cumulative Index to Nursing and Allied Health Literature (CINAHL) and the Scopus databases were searched up to September 2018 with the following MeSH terms and/or keywords combined with the Boolean operator AND/OR: (a) “Stroke”, “Delirium/diagnosis”, “Sensitivity and Specificity”, “Neuropsychological Tests”, “Validation Studies”, “Diagnostic test accuracy studies” in Medline database; (b) “Stroke”, “Delirium”, “Validation Studies”, “Neuropsychological Tests”, “Clinical Assessment Tools” in CINAHL database; and (c) “Stroke”, “Delirium”, “Screening Tools”, “Screening Tests”, “Validation Studies”, “Diagnostic test accuracy studies”, “Clinical Assessment Tools” in Scopus database. The reference lists of the retrieved studies were also screened for additional references, and studies that published findings of retrieved study protocols were all hand-searched.

### Inclusion and exclusion criteria

Eligible studies were those that satisfied the following inclusion criteria: (a) diagnostic test accuracy studies; (b) evaluating tools detecting delirium among patients with acute stroke; (c) written in English; and (d) published up to September 2018. Therefore, there were excluded articles: (a) reporting protocols regarding diagnostic test accuracy studies; (b) evaluating tools aimed at screening other cognitive issues in patients with acute stroke (e.g., dementia, cognitive decline); (c) analysing the association between post-stroke delirium and some risk factors or long-term consequences (e.g., dementia); and (d) not conducted in the acute phase of stroke, established as the first 48 h after the onset to the following two weeks [17].

### Study selection

**Fig. 1:**
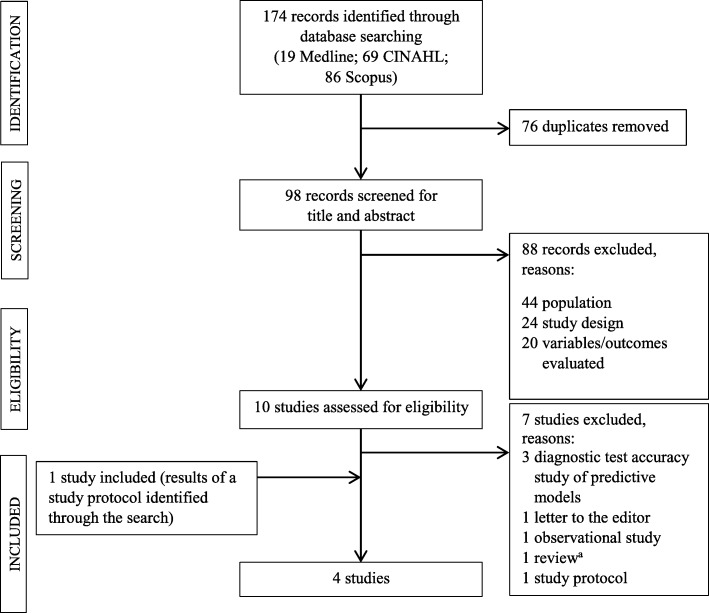
Flowchart for the search and study selection process (following the PRISMA guidelines) [15] *CINAHL* The Cumulative Index to Nursing and Allied Health Literature, *PRISMA* Preferred Reporting Items for Systematic Reviews and Meta-Analyses. ^a^no studies included in the review were pertinent to the inclusion criteria.

One researcher (IM) performed the literature search and two researchers (IM, AP) worked independently to evaluate study eligibility based on title and abstract screening of each study that emerged. Any differences in the evaluation regarding eligibility was discussed with a third researcher (LS). The full texts of eligible studies were then retrieved. Two researchers (IM, AP) independently evaluated each study by carefully reading the full text, and the study inclusion was decided upon joint agreement. The entire process of study inclusion is depicted in Fig. 1.

### Data extraction

Two researchers (IM, AP) extracted data from each included study and populated a study-specific pro forma reporting the following information: author; year of publication; study design; country; aim(s); the tool(s) that was validated and the language of validation; the gold standard or the alternative methods (e.g., informant interviews, other tools), if any, the tool was validated against; the diagnostic practices used in the data collection process, including the timeframe and the setting(s) where the data collection was performed; who assessed the patient with the tool(s) (e.g., neurologist, student); which population was the target and which patients were involved, by extracting the inclusion and the exclusion criteria; the sampling method and the main profile (age, gender) of patients included. Moreover, all diagnostic accuracy properties assessed were also checked and extracted, such as sensitivity, specificity, positive and negative predictive values, internal consistency, accuracy, interrater reliability, likelihood ratio and the Area Under the Curve (AUC).

On a preliminary fashion, the pro forma used has been piloted by analysing two studies; then, researchers worked independently and compared the data extracted from all studies. Differences were discussed with a third researcher (LS) and full agreement was reached.

Moreover, given the wide heterogeneity regarding the terminology used by studies as: screening, detection, assessment, diagnostic tool(a), all aimed at detecting early the phenomenon of interest to allow appropriate preventive or treatment interventions, we have used “delirium detection” as an overarching term.

### Quality evaluation of the study included

The included studies were evaluated in their quality by two researchers (IM, AP) using the Quality Assessment of Diagnostic Accuracy Studies-2 (QUADAS-2) [18]. Among different tools available, QUADAS-2 was chosen as precisely developed for systematic reviews of diagnostic test accuracy studies and recommended by the Agency for Healthcare Research and Quality, by the Cochrane Collaboration and by the UK National Institute for Health and Clinical Excellence [18, 19].

The tool is aimed at evaluating the “Risk of bias” that occurs if systematic flaws or limitations in (a) the patient selection, (b) the index test, (c) the reference standard used, as well as in (d) the flow and timing of the study, have threaten the findings [18]. The QUADAS-2 tool is also aimed at evaluating the “Applicability concerns”, as the extent to which the primary diagnostic test accuracy is applicable to the research question addressed by the systematic review. A limited applicability can be diagnosed as follows: (a) when, as compared with the review question, the study under evaluation has been conducted in patients with different demographic and/or clinical characteristics; (b) when the index test has been applied or interpreted differently; or (c) when it has been applied with a different definition of the target condition [18]. Each QUADAS-2 tool’s domain is composed of specific questions that can be answered as “yes” or “no” according to the evaluation performed by carefully reading the study. Moreover, the answer can also be “unclear” when the data available in the study under evaluation is not adequate.

## Results

### Characteristics of the studies included

A total of four diagnostic test accuracy studies were included in this review (Table 1), of which two [8, 21] had an additional observational phase and one [20] had an additional quasi-experimental phase. All studies were published from 2012 to 2017, three were conducted in Europe (Czech Republic [8]; Italy [20]; UK [22]) and one in Russia [21] and, therefore, validated in different languages.

**Table 1:** Main data extracted from the included studies

Author, year [reference]	Study design, Country	Aims	Evaluated tools, language	Gold standard/alternative methods(s) considered	Diagnostic practice	Examiner(s)	Patients: inclusion/exclusion criteria	Patients: sample methods and main profile
Infante et al., 2017 [20]	Diagnostic test accuracy study quasi-experimental study^a^ Italy	1. To assess the effect of DSM-V delirium criteria review and formal training on the ability of neurologists to recognise delirium 2. To evaluate the 4AT for the evaluation of post-stroke delirium	4AT Language: Italian	DSM-V criteria	Delirium was screened with 4AT and assessed with DSM-V criteria at admission and after 7 days of hospitalisation by the same researcher All diagnoses were afterwards reviewed independently by other two expert researchers Period: NR Setting: single tertiary stroke centre	Three neurologists	Inclusion criteria (diagnostic test accuracy study): • > 18 years • diagnosis of acute stroke • GCS > 5 Exclusion criteria: • aphasia • dementia	Consecutive sample *n* = 100; median age 79 years; gender NR
Kutlubaev et al., 2016 [21]	Diagnostic test accuracy study and observational study^a^ Russia	1. To identify older patients with high delirium risk 2. To assess the diagnostic value of the 4AT test in this population	4AT Language: Russian	DSM-IV criteria	Patients were examined for delirium within hours after their admission or on the next day; then twice at the interval of 12–24 h during their in-hospital stay Delirium was diagnosed according to the DSM-IV criteria and the 4AT test Period: 2 months (2013–2014) Setting: Neurovascular Department	Neurologist (not specified if the same, or not, who evaluated the delirium presence with both the 4AT and the DSM-IV criteria)	Inclusion criteria: • ≥ 65 years • admitted in the first 3 days of stroke Exclusion criteria: • subarachnoid/subdural haemorrhages without intracerebral haematoma • transient ischaemic attacks • impairment of consciousness as severe as sopor and coma • with significant chronic mental disorders in the past	Consecutive sample *n* = 73 (over 132 eligible); median age 79 years; male 29%
Lees et al., 2013 [22]	Diagnostic test accuracy study United Kingdom	1. To describe test accuracy properties of various brief screening assessments against an independent clinical diagnosis of cognitive impairment (using MoCA) and delirium 2. To describe the effect of altering the screen-positive cut-point for MoCA using differing predetermined diagnostic thresholds	AMT-10 AMT-4 CDT COG-4 4AT GCS Single Question “Does this patient have cognitive issues?” at the daily multidisciplinary team Language: English	CAM	Patients were assessed during the period of day 1 to day 4 after stroke unit admission Period: 10 weeks (April–June 2012) Setting: Stroke Unit	Two trained medical students: one completed the delirium assessment using the validating tools; one assessed for delirium using the CAM They were blinded	Inclusion criteria: • cerebral ischaemia and haemorrhage • medically stable to allow an attempt at a least part of cognitive assessment Exclusion criteria: NR	Consecutive sample *n* = 111 (over 138 eligible); median age 74 years; male 50%
Mitasova et al., 2012 [8]	Diagnostic test accuracy study and observational study^a^ Czech Republic	1. To describe the epidemiology of delirium in a cohort of acute post-stroke patients using the DSM-IV 2. To determine the sensitivity, specificity, and overall accuracy of the CAM-ICU, and 3. To investigate its validity as a routine monitoring instrument for hospitalised patients with stroke by non-psychiatrically trained clinicians	CAM-ICU Language: Czech	DSM-IV criteria	Patients underwent paired daily evaluation with the CAM-ICU The first CAM-ICU evaluation on the first day after stroke onset and admission (day 1) and then daily (6 days/week) on at least 7 consecutive days on which the patient was accessible to testing (RASS ≥ −3). If delirium was present on day 6 or 7, its assessment follow-up continued until at least 2 subsequent days without delirium were recorded In patients with consciousness deterioration the follow-up was stopped The standard DSM evaluation of delirium was performed < 2 h apart daily Period: 18 months (2009–2010) Setting: specialised stroke centre	A trained junior physician assessed patients with the CAM-ICU A panel of specialists, experts on delirium (two neurologists, two neuropsychologists, a psychiatrist and a speech therapist) performed the standard reference DSM evaluation (at least one neurologist and one neuropsychologist)	Inclusion criteria: • cerebral infarction or intracerebral haemorrhage • delirium assessment within 24 h of stroke onset • approval of the patient or his or her relatives Exclusion criteria: • patients who did not speak Czech • duration of stroke symptoms and signs < 24 h • history of severe head trauma or neurosurgery (at any time) • subarachnoid haemorrhage, venous infarction, brain tumour • history of psychosis • patients who were comatose or stuporous on admission and did not improve during the first week post-stroke (RASS ≤ − 4)	Consecutive sample *n* = 129 (151 initially enrolled, over 331 eligible); mean age 71.3 years; male 55.8%

*4AT*: 4-Assessment Test for delirium, *AMT:* Abbreviated Mental Test, *CAM:* Confusion Assessment Method, *CAM-ICU:* Confusion Assessment Method for the Intensive Care Unit, *CDT:* Clock Drawing Test, *COG4*: Cognitive examination derived from National Institutes of Health Stroke Scale (NIHSS), *DSM*: Diagnostic and Statistical Manual of mental disorders, *GCS*: Glasgow Coma Scale, *MoCA*: Montreal Cognitive Assessment, *NR* not reported, *RASS* Richmond Agitation and Sedation Scale.

^a^only data regarding validation phase has been extracted and reported in this Table

The included studies developed diagnostic test accuracy evidence regarding the following tools: the 4-Assessment Test for delirium (4AT) [20–22], the Confusion Assessment Method for the Intensive Care Unit (CAM-ICU) [8], the Abbreviated Mental Test, in the complete and in the short version (AMT-10 and AMT-4) [22], the Clock Drawing Test (CDT) [22], the Glasgow Coma Scale (GCS) [22], and the Cognitive examination (COG4) [22]. The following gold standards/alternative tools were considered in the validation processes undertaken: three studies used the DSM criteria [8, 20, 21] and one used the CAM [22].

Moreover, delirium detection was performed mainly by neurologists, neuropsychiatrists or physicians. Mitasova et al. [8] involved a panel of specialists who were considered “delirium experts” for the reference evaluation of delirium. However, the assessment was not always detailed if it was blinded between the tool under study and the gold standard/alternative method(s) or if the assessment was performed by one or several researchers [20, 21].

With regards to the timeframe, Infante et al. [20] used a single evaluation of delirium, while the remaining studies [8, 21, 22] performed several assessments; the follow-up lasted from four days after hospital admission [22] to the entire length of stay in the hospital [21].

All studies adopted a consecutive sample method using heterogeneous inclusion criteria. All included patients with both ischaemic and haemorrhagic stroke and with a certain degree of consciousness as evaluated by the clinical team or using the GCS score > 5. However, Infante et al. [20] excluded patients with aphasia and dementia, while Mitasova et al. [8] and Kutlubaev et al. [21] also excluded patients with a history of severe head trauma, neurosurgery, brain tumour or significant chronic mental disorders/psychosis.

The study sample sizes were in general small (from 73 to 129 participants), with an age ranging from 71.3 to 79 years. Moreover, all studies were monocentric in nature and performed in hospital units, mainly in Stroke Units/Stroke centres [8, 20, 22].

### Quality evaluation of the studies included

As shown in Table 2, all studies reported a low risk of concerns in the “Applicability concerns” domain. Conversely, among the “Risk of bias”, the “Patient selection” and the “Reference standard” domains, major concerns have emerged, respectively due to: the exclusion of patients with specific characteristics that have potentially affected the representativeness of the sample as, for example, dementia that has been documented to raise the risk of delirium [4], and the use of a gold standard tool to detect delirium not previously validated in patients with stroke [22]. Moreover, in the same domain, with regards to the study performed by Infante et al. [20] some concerns emerged regarding the examiner’s knowledge of the results obtained with the tools under evaluation when assessing delirium with the gold standard. Kutlubaev et al. [21] were instead evaluated for unclear risks in the “Index test” evaluation as it was not possible to assess if the index test findings were interpreted without knowledge of the findings of the reference standard, or not.

**Table 2:** Quality evaluation of the included studies according to the QUADAS-2 [18]

Study, author, year [reference]	Risk of Bias										Applicability Concerns		
	Patient Selection			Index Test		Reference Standard		Flow and Timing			Patient Selection	Index Test	Reference Standard
	Was a consecutive or random sample of patients enrolled?	Was a case-control design avoided?	Did the study avoid inappropriate exclusions?	Were the index test results interpreted without knowledge of the results of the reference standard?	If a threshold was used, was it pre-specified?	Is the reference standard likely to correctly classify the target condition?	Were the reference standard results interpreted without knowledge of the results in the index test?	Was there an appropriate interval between the index test and reference standard?	Did all patients receive the same reference standard?	Were all patients included in the analysis?	Are there concerns that the included patients and setting do not match the review question?	Are there concerns that the index test, its conduct, or its interpretation differ from the review question?	Are there concerns that the target condition as defined by the reference standard does not match the question?
Infante et al., 2017 [20]	Y	Y	N	N	Y	Y	N	Y	Y	Y	N	N	N
Kutlubaev et al., 2016 [21]	Y	Y	Y	?	Y	Y	?	Y	Y	Y	N	N	N
Lees et al., 2013 [22]	Y	Y	N	Y	Y	?	Y	Y	Y	Y	N	N	N
Mitasova et al., 2012 [8]	Y	Y	N	Y	Y	Y	Y	?	Y	Y	Y	N	N

*Y*: yes, *N*: no,?: unclear (not enough data documented in the study), *QUADAS-2*: Quality Assessment of Diagnostic Accuracy Studies-2.

All studies reported a low risk of bias for the “Flow and timing” evaluation, and only Mitasova et al. [8] reported an unclear risk of bias because the appropriateness of the interval between the index test and the reference standard was not documented in the article.

### Accuracy properties of the tools

As shown in Table 3, for the 4AT, the studies reported a sensitivity range of 90.2–100%, a specificity range of 64.5–86%, a positive predictive value range of 43–86% and a negative predictive value range of 85.6–100% [20–22]. Moreover, the AUC was evaluated only for the 4AT, ranging from 0.82 to 0.89 [20, 21].

**Table 3:** Delirium detection tools emerged from the included studies: comparison in the diagnostic test accuracy properties documented

Tool	Author, year [reference]	Sensitivity % (95% CI)	Specificity % (95% CI)	Positive predictive value % (95% CI)	Negative predictive value % (95% CI)	Internal consistency (Cronbach α)	Accuracy % (95% CI)	Interrater reliability (Ƙ)	Likelihood ratio	AUC	Gold standard considered
4AT	Infante et al., 2017 [20]	90.2^b^	64.5^b^							0.82^a^	DSM-V criteria
		96.4^c^	76.7^c^							0.88^b^	
	Kutlubaev et al., 2016 [21]	93	86	86	85.6	0.80				0.89	DSM-IV criteria
	Lees et al., 2013 [22]	100 (74–100)	82 (72–89)	43	100						CAM
		100^a^ (72–100)	83^a^ (73–88)								CAM
CAM-ICU	Mitasova et al., 2012 [8]	76 (55–91)	98 (93–100)	91 (70–99)	94 (88–98)		94^d^ (88–97)	0.94 (0.83–1.00)	0.47 (0.27–0.83)		DSM-IV criteria
AMT-10	Lees et al., 2013 [22]	75 (43–95)	61 (51–71)	21	95						CAM
AMT-4	Lees et al., 2013 [22]	83 (52–98)	61 (51–71)	23	96						CAM
CDT	Lees et al., 2013 [22]	67 (22–96)	38 (28–49)	7	95						CAM
COG4	Lees et al., 2013 [22]	70 (35–93)	44 (35–55)	13	92						CAM
GCS	Lees et al., 2013 [22]	17 (2–48)	81 (71–88)	11	88						CAM
“Does this patient has cognitive issues?“^e^	Lees et al., 2013 [22]	58 (28–85)	85 (76–92)	35	93						CAM

^a^subgroup analysis, excluding patients with severe aphasia (n = 7; over 111 patients included); ^b^at the admission; ^c^after 7 days; ^d^considered as ratio: true-positives + true-negatives/true positives + false-positives + true-negatives + false-negatives; ^e^the single question was asked to healthcare professionals of the multidisciplinary team

*4AT*: 4-Assessment Test for delirium, *AMT*: Abbreviated Mental Test, *AUC* Area Under the Curve, *CAM*: Confusion Assessment Method, *CAM-ICU:* Confusion Assessment Method for the Intensive Care Unit, *CDT*: Clock Drawing Test, *CI*: Confidence of Interval, *COG4*: cognitive examination derived from National Institutes of Health Stroke Scale (NIHSS), *DSM*: Diagnostic and Statistical Manual of mental disorders, *GCS*: Glasgow Coma Scale.

The CAM-ICU [8] reported a sensitivity of 76% (95% Confidence Interval [CI] 55–91), a specificity of 98% (95% CI 93–100), a positive predictive value of 91% (95% CI 70–99) and a negative predictive value of 94% (95% CI 88–98). Additionally, Mitasova et al. [8] also estimated that the tool accuracy was 94% (95% CI 88–97) and had an inter-rater reliability (Ƙ) of 0.94 (95% CI 0.83–1.00), with a re-analysis of ten delirium evaluations as previously video-taped by an expert, and a likelihood ratio of 0.47 (0.27–0.83).

Lees et al. [22] evaluated the properties of several tools, in which the AMT-10 reached a sensitivity of 75% (95% CI 43–95) and a specificity of 61% (95% CI 51–71), while the AMT-4 obtained a sensitivity of 83% (95% CI 52–98) and a specificity of 61% (95% CI 51–71). In the same study, the single question “Does this patient have cognitive issues?” asked to the healthcare professionals of the multidisciplinary team reached a sensitivity of 58% (95% CI 28–85) and a specificity of 85% (95% CI 76–92). A detailed report of the accuracy properties has been shown in Table 3.

## Discussion

We have performed a systematic review of diagnostic test accuracy studies regarding the detection of delirium among patients with acute stroke. An accurate early detection of delirium might provide opportunities to identify high risk patients, to implement evidence-based interventions designed to prevent or minimize delirium occurrence, as well as to protect patients against the consequences of delirium. Moreover, having accurate tools might also (a) reduce false positive detection, which may increase the confidence and the following use of the tool by clinicians, (b) reduce costs in the form of increased surveillance, as well as (c) reduce harm, as for example, the burden of the families called to stay at the bedside when their beloved is detected at high risk of delirium.

With regards to the first aim of this review, despite stroke patients being particularly vulnerable to delirium [5, 6] due to direct cerebral insult [23] and the presence of several risk factors, such as cognitive, visual and functional impairments [6] with an incidence ranging from 13 to 48% [24], only four studies to date, mainly across Europe, have investigated how to detect the delirium in these patients. Research validating delirium detection tools in patients with acute stroke has only started recently. As reported by Carin-Levy [13], no diagnostic test accuracy studies have been conducted before 2010 and all studies included in our review [8, 20–22] were published between 2012 and 2017, suggesting an increasing interest in this research field.

Concerning the quality of research studies available, some methodological limitations have emerged suggesting that further research should follow available guidelines (e.g., QUADAS-2, [18]) and assess a minimum set of accuracy properties allowing a full comparison across tools. Moreover, in available studies, tools have been used by physicians, medical students or unspecified healthcare professionals of a multidisciplinary team—suggesting that there is a greater source of heterogeneity in their validation regarding the health care professional profile. Future studies should also involve clinical nurses given their presence at the bedside 24/7 who can develop an in-depth knowledge regarding the cognitive status of the patients. Furthermore, relatives are often involved in daily practice as a reference point regarding the incurred changes in cognition as compared with the pre-stroke daily life, by detecting weak changes given their familiarity with the patient: therefore, further studies should also consider involving family members in the evaluation of the tool’s accuracy. Their involvement can also increase (a) the duration of studies and their likelihood to be performed across settings (from acute to post-acute care), as well as (b) the quality of studies when family members are involved in the assessment regarding the prior clinical conditions (e.g., if patient reported, or not, delirium episodes before the acute stroke). Moreover, studies performed on this topic focused their attention on the acute phase of stroke, with assessments not lasting until the resolution of the delirium condition. Without long-term follow-ups, the strength of delirium condition detection could be uncertain and affect the accuracy properties of the tools.

A total of seven tools detecting delirium have been validated to date among patients with acute stroke. However, more than 20 tools have been developed and/or validated in other fields, such as Intensive Care Units (e.g., CAM, CAM-ICU), surgery (e.g., CAM, Delirium Detection Score, Nurses Delirium Screening Checklist, Delirium Observation Screening Scale), emergency unit (brief-CAM, Delirium Triage Screen) and oncology/palliative care (e.g., CAM-ICU, Intensive Care Delirium Screening Checklist) [25, 26]. Several limitations have been reported among these studies [26] suggesting that in the field of neuropsychological tests researchers encounter great challenges.

The 4AT is the most validated tool in patients with acute stroke, with studies reporting good accuracy properties [20–22]. Having a tool with high sensitivity is recommended in the available literature [27] given that it can ensure an early detection of delirium which has been documented to be largely underestimated among patients with acute stroke [20]. Moreover, although a high sensitivity could lead to an overestimation of the phenomenon [20], the 4AT can allow immediate intervention at the onset of delirium, potentially preventing or minimizing the consequent negative outcomes. The AUC, calculated only for the 4AT [20, 21], ranged from 0.82 to 0.89, thus, confirming the accuracy of the tool [28] —with a good internal consistency as measured with the Cronbach alpha [20]— that suggests the items included measure the same construct.

According to previous studies in the field, the 4AT is a rapid tool, easy to use for all healthcare professionals without specific training [25]. In addition, it has been documented to be adequate for screening patients with fluctuating levels of consciousness and drowsiness, and appears to be useful not only in hyperactive but also in hypoactive delirium [25], which has been widely reported as being underdiagnosed [14, 29]. Additionally, despite most delirium detection tools requiring a verbal response, the 4AT properties have been reported to not change significantly in patients with acute stroke and severe aphasia [22]. Furthermore, the 4AT has been reported to be adequate in the complex identification of delirium superimposed on dementia [25].

The CAM-ICU has been validated in patients with acute stroke only by Mitasova et al. [8]. Compared to the 4AT, the CAM-ICU demonstrated an inferior sensitivity and a higher specificity. Likewise, the 4AT has been reported to have good accuracy and a high inter-rater reliability across examiners, properties that cannot be compared with other tools given that these properties have only been provided for the CAM-ICU. The Likelihood Ratio data is moderate, thus, suggesting that the tool is not reliable in detecting the absence of delirium.

The CAM-ICU was originally developed for delirium detection among critically ill patients, specifically those mechanically ventilated [30, 31]. Subsequently, the CAM-ICU tool has been widely validated in emergency, oncology, palliative and surgical patients [25], which are all considerably different than stroke patients. The sensitivity and specificity properties have been reported to be influenced by the health care professional’s experience and training [25], thus, suggesting that the tool requires training [25, 26] to maximise its accuracy [25]. In contrast to the 4AT, the CAM-ICU allows a limited assessment of drowsy patients [25], with a potential risk of underdiagnosing those with hypoactive delirium.

Other delirium detection tools have been validated by Lees and colleagues [22], such as the AMT-10, the AMT-4, the CDT, the COG4 and the GCS. All reported lower psychometric properties as compared with the 4AT and the CAM-ICU, likely because these tools were not specifically designed for detecting delirium. In fact, the AMT-10 and its shortened form, AMT-4, allows a direct cognitive test of mental impairment [32, 33]. The CDT [34] does not require a verbal response from the patient, thus, it can be suitable for stroke patients [22], but it has been shown to be useful in cognition measurement—not in delirium detection [26]. Similarly, the COG4 is derived from the National Institute of Health Stroke Scale [35] and adequately designed to evaluate the cognitive status in patients with acute stroke [35]. The issue is the same regarding the GCS [36], which has been designed to assess coma and impaired consciousness. Delirium could be a consequence of post-stroke cognitive impairments [22], but the use of the above-mentioned tools in detecting delirium among these patients should be considered with caution.

To date only Lees et al. [22] have tested some properties of a single question “Does this patient have cognitive issues?”, which is often used in clinical practice to detect changes in the patients’ cognition by also interviewing family caregivers. Data from their study suggested that this single question has comparable specificity to other structured tools (e.g., 4AT, CAM-ICU) and superior sensitivity with regards to the CGS.

A further consideration is needed regarding the gold standard or the alternative measures criteria used across studies. Infante et al. [20], Kutlubaev et al. [21] and Mitasova et al. [8] considered the DSM-IV criteria as a point of reference for the 4AT and the CAM-ICU, respectively. The DSM-IV criteria could be considered as a gold standard, but need to be performed by experts in the field, such as neurologists or psychiatrists, and requires special training [21]. Alternatively, Lees and colleagues [22] assessed the 4AT properties against the CAM, although this tool has not been validated in delirium detection among patients with acute stroke.

Therefore, more studies are encouraged in this field to establish the accuracy of the tools detecting delirium among patients with acute stroke; alongside, these tools should be considered inside of the widely clinical examination of the patients [37].

### Strengths and limitations

This systematic review offers an overview of the delirium detection tools available in the literature regarding patients with acute stroke, in which the assessment could be a challenge due to specific neurological symptoms (e.g. aphasia). Accuracy properties of different tools have been analysed, as well as the methodological quality of the studies documenting these properties. However, this review is affected by several limitations. Firstly, we did not register and publish the review research protocol in a public access repository. Moreover, only Medline, CINAHL, and Scopus databases were searched and only studies published in English and peer reviewed were included, thus introducing a potential source of selection bias. Authors were not contacted to obtain missing or incomplete data that was not reported in the included studies due, for example, to word restrictions imposed by journal guidelines, and this could have introduced information bias and affect the completeness of the quality evaluation as performed with the QUADAS-2 [18].

Some accuracy properties were heterogeneously reported across studies thus threatening a full comparison of the findings: for example, 95% CI has been not reported in Kutlubaev et al. (2016) [18] or in Infante et al. (2017) [20]; moreover, sensitivity and specificity have all been reported while positive and negative predictive values were not. Attempts were performed to calculate the missing metrics, but no reliable findings were obtained due to some missing data in the studies: this strengthens the need to further adopt guidelines [18] in reporting test accuracy studies regarding delirium.

## Conclusions

The detection of delirium in stroke is complex, given the challenges of (a) the high occurrence of sensory, perceptual and communication deficits with stroke, (b) the differentiation of delirium from more permanent stroke-related acquired cognitive impairments, and (c) the pre-existing cognitive impairments, given that vascular disease and stroke are risk factors for both dementia and further stroke. However, having instruments supporting the clinical judgment is crucial to personalise pharmacological and non-pharmacological interventions [38, 39] preventing and/or managing this clinical condition and its negative consequences.

Despite its relevance, to date few primary studies have been published to test accuracy properties of tools in their ability to detect post-stroke delirium; among those available, the 4AT and the CAM-ICU tools have been mostly studied. However, the small number of studies retrieved have documented different measures thus preventing comparisons across tools and suggesting that research has just started to add evidence to the challenge of detecting and usefully assessing newly-acquired delirium in stroke disease. Furthermore, without long-term follow-ups at least until the resolution of the delirium, it is uncertain how robust the diagnosis of delirium can be. Therefore, further studies methodologically sound and reporting a minimum data set of metrics in order to ensure comparison across studies, are strongly recommended. Moreover, alongside the importance of simple tools in the detection of newly-acquired delirium, a full consideration of the use of structured informant histories from either family or nurses and other care staff should be considered.

## Data Availability

The datasets used and/or analyzed during the current study are available from the corresponding author on reasonable request.

## Abbreviations

4AT: 4-Assessment Test for delirium
AMT: Abbreviated Mental Test
AUC: Area Under the Curve
CAM: Confusion Assessment Method
CAM-ICU: Confusion Assessment Method for the Intensive Care Unit
CDT: Clock Drawing Test
CI: Confidence Interval
CINAHL: The Cumulative Index to Nursing and Allied Health Literature
COG4: Cognitive examination derived from National Institutes of Health Stroke Scale (NIHSS)
DSM: Diagnostic and Statistical Manual of mental disorders
GCS: Glasgow Coma Scale
MoCA: Montreal Cognitive Assessment
N: No
NR: Not reported
PRISMA: Preferred Reporting Items for Systematic Reviews and Meta-Analyses
QUADAS-2: Quality Assessment of Diagnostic Accuracy Studies-2
RASS: Richmond Agitation and Sedation Scale
Y: Yes? Unclear (not enough data)
